# Cognitive impairment in psychoses and affective disorders: addressing future challenges

**DOI:** 10.1192/j.eurpsy.2025.102

**Published:** 2025-08-26

**Authors:** G. Sachs, A. Erfurth

**Affiliations:** 1 Medical University of Vienna; 2 1st Department of Psychiatry and Psychotherapeutic Medicine, Klinik Hietzing, Vienna, Austria

## Abstract

**Abstract:**

Cognitive dysfunction is well documented in patients with schizophrenia and bipolar disorder, including impairments in attention, memory, executive function and social cognition. These impairments are associated with poor social functioning and reduced activities of daily living.

Various therapeutic interventions have targeted cognitive impairment in both schizophrenic and bipolar patients. Several trials and meta-analyses are currently available. In addition to psychopharmacology, cognitive remediation programmes are available and have been shown to be applicable in clinical practice.

In conclusion, as we move towards more integrative and personalised treatment strategies for schizophrenic and bipolar patients, new assessments and therapies are available to target cognitive dysfunction. Not least because there are currently no cognitive enhancers on the market, it is hoped that new pharmacological adjunctive strategies can improve cognition in schizophrenia and bipolar disorder.

**Disclosure of Interest:**

None Declared

